# Evolution of documents related to strength training research on competitive swimmers: a bibliometric review

**DOI:** 10.3389/fspor.2025.1603576

**Published:** 2025-06-19

**Authors:** Tiago F. Venâncio, Mário J. Costa, Catarina C. Santos, Nuno Batalha, Víctor Hernández-Beltrán, José M. Gamonales, Mário C. Espada

**Affiliations:** ^1^ SPRINT Sport Physical Activity and Health Research & Innovation Center, Centro de Investigação e Inovação em Desporto Atividade Física e Saúde (SPRINT), Rio Maior, Portugal; ^2^ Training Optimization and Sports Performance Research Group (GOERD), Faculty of Sport Science, University of Extremadura, Cáceres, Spain; ^3^ Life Quality Research Centre (CIEQV), Instituto Politécnico de Setúbal, Santarém, Portugal; ^4^ Biosciences Higher School of Elvas, Polytechnic Institute of Portalegre, Elvas, Portugal; ^5^ Centre of Research, Education, Innovation, and Intervention in Sport (CIFI2D) and Porto Biomechanics, Laboratory (LABIOMEP-UP), Faculty of Sport, University of Porto, Porto, Portugal; ^6^ Higher Education School (ESEC), Polytechnic of Coimbra, Coimbra, Portugal; ^7^ Department of Sport Sciences, Exercise and Health, University of Trás-os-Montes and Alto Douro (UTAD), Vila Real, Portugal; ^8^ Departamento de Desporto e Saúde, Escola de Saúde e Desenvolvimento Humano, Universidade de Évora, Évora, Portugal; ^9^ Comprehensive Health Research Centre (CHRC), Universidade de Évora, Évora, Portugal; ^10^ Faculty of Education and Psychology, University of Extremadura, Badajoz, Spain; ^11^ Instituto Politécnico de Setúbal, Escola Superior de Educação, Setúbal, Portugal; ^12^ Centre for the Study of Human Performance (CIPER), Faculdade de Motricidade Humana, Universidade de Lisboa, Lisbon, Portugal

**Keywords:** research, trends, swimming, scientific literature, academic production

## Abstract

**Background:**

The purpose of this study is to conduct a bibliometric and scoping review to map the evolution of scientific literature on strength training in competitive swimmers.

**Methods:**

Web of Science database was used considering the time frame between 1980 and 2024. The following terms were used to search documents: “swimmers”, “strength”, and “training”. A total of 460 documents were included for statistical analysis

**Results:**

Until 2000, fewer than 5 manuscripts were published annually, and only after 2011 did the number regularly exceed 20, peaking in 2021 with 43 publications. Among the top 10 authors, 7 are Portuguese, each with at least 7 published papers. A total of 726 institutions were identified, with 29 having published at least 5 studies. The “Universidade da Beira Interior” had the most publications (20), while the “University of Copenhagen” received the most citations (619) with just 3 papers. Sixty countries contributed overall, with 10 publishing at least 20 documents; the United States of America led with 83. The most prominent keywords over time were “sports performance,” “speed,” and “swimming performance.”

**Conclusion:**

These results highlight that bibliometric analysis provides pertinent information, very useful regarding research trends and networks, aiming for future research on the topic of strength in swimmers.

## 1 Introduction

Swimming is the second most popular sport in the Olympic Games based on the number of athletes (1). The goal in competitive swimming is to cover a given distance in the shortest possible time (2) and these days, due to the increased professionalization of the sport, it has become increasingly difficult for amateur athletes to reach finals or medal positions at the international level (3). As the level of performance has continuously improved, competition results reflect this shift. Sports performance has been enhanced from decade to decade, breaking previous records and reaching previously unreachable performances recently (4, 5).

A podium finish in swimming competitions can be separated by a 100 ms time difference (6), and dry-land performance has been shown to contribute to overall swim performance in different events (7). Human locomotion in water results from the interaction of propelling limbs with the fluid. Swimmers’ capacity to move through the water depends on the amount of applied propulsive force and on the drag forces opposed to a forward motion (8). Swimming performance is related to a deterministic model since strength will influence the force, being this determined by kinematics, which in turn is influenced by anthropometrics, which in turn, influences the kinetics (9–12).

Since the early 20th century, there has been discussion over the significance of strength or strength training (ST) for swimming performance. This was linked to Robert Kiphuth, who was probably among the first swimming instructors in the 1920s and 1930s to use dry-land training—training outside of a pool to build the muscles necessary for swimming performance (13). The ST is one method of training that helps athletes improve their muscular force–velocity function and ability to enhance endurance athletes' performance through neuromuscular adaptations, increasing economy and endurance-specific muscle power components (14), increasing the rate at which force is produced, which leads to improved performance (15). Improvements in swimming sprint performance ranging from 1.3% to 4.4% have been documented when dry-land ST is used (16).

To reduce water resistance in short race distances, research has specifically concentrated on the impact of force production and strength on speed enhancement (7). Thus, it is important to carry out programs to promote ST in swimmers and evaluate the programs and their impact on the swimmers' profiles and performance. This demonstrates a steadfast commitment to learning and a noteworthy contribution to literature. Previously, Veiga and Roig (17) pointed out that underwater phases and stroke efficiency are important factors that determine success, which emphasizes the need for ST in swimming. Stronger core, lower, and upper body muscles enable swimmers to exert more force during each stroke's pull phase, increasing stroke efficiency and distance per stroke, according to scientific research (18). In a similar vein, strong leg muscles help produce stronger, quicker kicks, which are essential for sustaining high water velocity (19). Swimmers can improve propulsion and body positioning by overcoming hydrodynamic drag more successfully with both strength and power (20). Furthermore, higher muscular power improves the swimmer's capacity to maintain high kick frequencies and stroke rates, both of which are essential for sustaining top speed during the sprint (7).

However, when ST is paired with aerobic stimulus, which is frequently used in aquatic sports training, these improvements appear to be adversely damaged (21). Studies to ascertain if ST improves swimming competition performance are scarce (22), while several studies have shown that muscle strength is strongly linked to performance in short- and high-intensity efforts; swimmers with greater strength also performed better over longer distances (21). According to some previous studies, strength plays a crucial part in swimming since it increases a swimmer's ability to generate force against water resistance, which in turn improves their speed and, eventually, their swimming performance (23).

A significant body of study in sprint swimming has surfaced intending to comprehend the critical performance components. Research has specifically examined the impact of force output and strength on speed development to overcome the water resistance in short race distances (24). Dry-land ST effect on performance, however, varies depending on the kind of training and adaptations made in addition to the exercises performed. Low-volume, high-force, or high-velocity resistance training regimens are advised for the best transfer to sprint performance (25).

A methodical approach to evaluating vast amounts of scientific data, bibliometric reviews provide important insights into research topics and future directions (26). By carefully analyzing publication patterns, fundamental contributors, and emerging clusters in the step test literature, they offer a unique perspective. They also identify the nations, journals, institutions, and authors that are most active in each field of study, as well as the current partnerships between authors, institutions, and nations (27). Through the review of abstracts obtained from the analysis of numerous articles from the past to the present, bibliometric studies allow researchers to effectively understand literature (28). Research on swimming competitions can reveal networks of collaboration among scientists, institutions, and countries. As far as we are aware, no bibliometric research has been done on strength training for competitive swimmers; this information is useful in understanding the global scientific collaboration on this subject (29). Therefore, the purpose of this study was to conduct a bibliometric and scoping review to map the evolution of scientific literature on strength training in competitive swimmers.

## 2 Materials and methods

### 2.1 Study design

The main objective of the present work was to develop a bibliometric review of the existing literature, which is framed within the Theoretical Studies (30). In addition, due to the evaluation of existing information, it is classified within the studies with retrospective methodology (31). This method allows obtaining more information related to the research topic, as well as understanding the state of the art (32). In the same way, it allows collecting documents from the same database, as well as filtering and refining the search simply (33). Therefore, the following phases were followed for the elaboration of the present study: (1) selection of the topic, (2) selection of the research method, (3) collection of information, (4) analysis of the information, (5) visualization, and (6) interpretation.

The analysis of scientific production regarding the effect of the ST on swimmers between 1980 and June of 2024 was conducted using bibliometrics as a research technique with the assistance of the Web of Science (WoS) database (34, 35).

### 2.2 Search strategy

The following terms were used to search documents: “swimmers”, “strength”, and “training”, using the following search equation (“swimmers AND strength AND training”) using the WoS database. Firstly, J.M.G. and V.H.B. carried out the search and selection of the documents. Secondly, if there was any disagreement, M.C.E. and T.F.V. discussed whether the study was valid and reliable to be selected for the bibliometric analysis.

The following inclusion criteria were considered: (1) Published articles (e.g., abstracts, presentations, not considered); (2) Documents related to competitive swimmers; (3) Published in English-language journals; and (4) Articles published up to June 2024. Exclusion criteria were: (i) articles written in languages other than English; (ii) articles lacking information related to competitive swimmers; and (iii) articles not retrieved or not retrieved from WoS. All the documents identified were included in the analysis to analyze the co-authorship of the studies regarding different variables. Finally, a total of 460 documents were included.

### 2.3 Data extraction

For the selection and extraction of information, the Web of Science (WoS) database was used, since it allows obtaining information related to the title, name of the publication (journal), year, abstract, and/or keywords. For this reason, it is one of the most widely used databases for the development of these works (36, 37). Specifically, those documents indexed in the WoS Core Collection database were considered, a series of documents only indexed in this electronic platform. VOSviewer software was used to carry out the analysis, create, and visualization of the figures aiming to analyze keyword co-occurrence, author collaboration networks and other relevant information (38).

### 2.4 Data analysis

For the statistical analysis, the most relevant laws of bibliometric reviews were considered (39). To evaluate the exponential growth of the selected documents, Price's law (40) was used through the calculation of the coefficient *R*^2^. In this way, the trend in the increase in the number of documents published concerning the topic was deducted. In addition, two time zones were established to categorize the documents in terms of year of publication: older documents, as the documents published before the median of the sample, which was 2009, and newer manuscripts, for those documents published in or after 2010.

To identify those authors with the largest output of published research documents on the topic, the Lotka analysis (41) was carried out, extracting the H-Index for each author identified in the search (42). All the authors of each study were extracted and analyzed, independently of the position they had in the list of authors consigned in the research document. The H-index was defined as the maximum value of *h*, such that the given author has published at least *h* papers that have each been cited at least *h* times (43). The H-Index was employed to identify those authors with higher potential contribution to the field under scrutiny (44). Additionally, the affiliations of each author were also collected and meta-analyzed to detect organizations with the highest research output on the topic.

In addition, to analyse the keywords most used by the authors in each of the papers (*n* = 372), Zipf's law was used (45, 46). Finally, for data analysis and visualization, Microsoft Excel (2006 version: Microsoft Corporation, Redmond, WA, USA) and VOSviewer (v.1.6.19 for macOS, Center for Science and Technology Studies, Leiden, The Netherlands) were used. For the creation and visualization of the results, a fragmentation analysis was used (attraction: 3 and repulsion: −3), depending on the theme and the temporality of the results (47).

## 3 Results

### 3.1 Evolution of the number of documents

**Figure 1:**
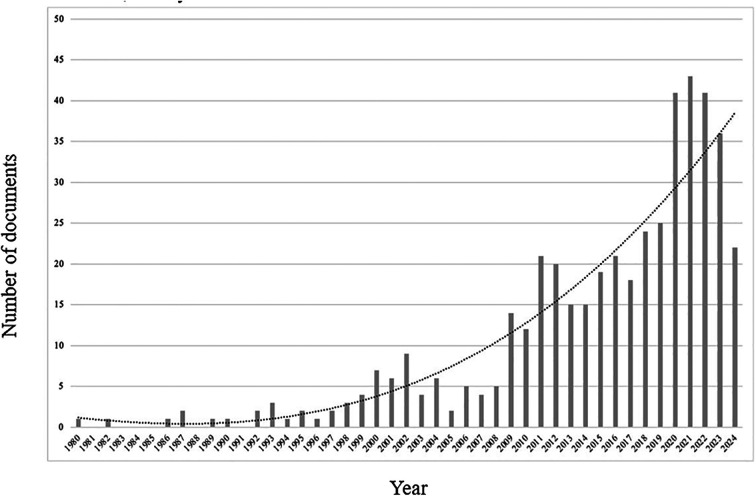
Evolution of the number of documents published by year between 1980 and 2024.

Bearing in mind the number of publications since the beginning in 1980, there is no continuity until 1992, with at least one document published per year until 2024, in which 2021 was the period with the highest number of published studies (*n* = 43), followed by 2020 and 2022, each year with 41 studies (Figure 1).

### 3.2 Interactions between the authors

**Figure 2:**
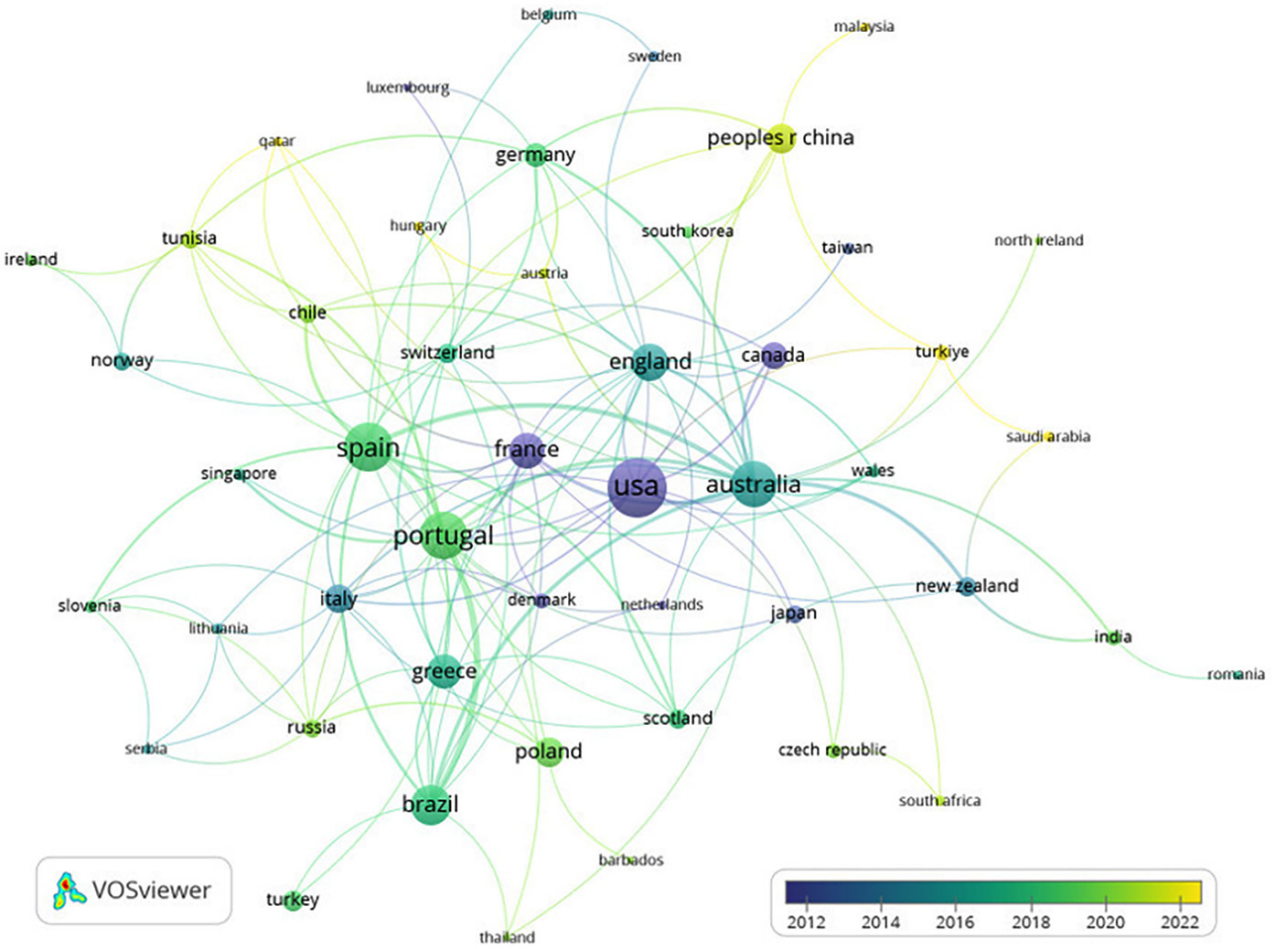
Node map for the relationship between the co-authorships.

Table 1 shows the most prolific authors, seven were identified with a minimum of 10 published manuscripts, with Marinho, D.A., associated with the largest number, with 17. In this line, Figure 2 shows the interactions between the most prolific authors regarding the study's aims.

**Table 1:** Top 10 authors associated with the publication of manuscripts within the ST topic.

Authors	Documents	% of 460
Marinho, D.A.	17	3.69
Barbosa, T.M.	13	2.82
Silva, A.J.	13	2.82
Pyne, D.B.	12	2.60
Toubekis, A.G.	11	2.39
Arellano, R.	10	2.17
Fernandes, R.J.	10	2.17
Batalha, N.	8	1.73
Costa, M.J.	7	1.52
Marques, M.C.	7	1.52

### 3.3 Interactions between the institutions

**Figure 3:**
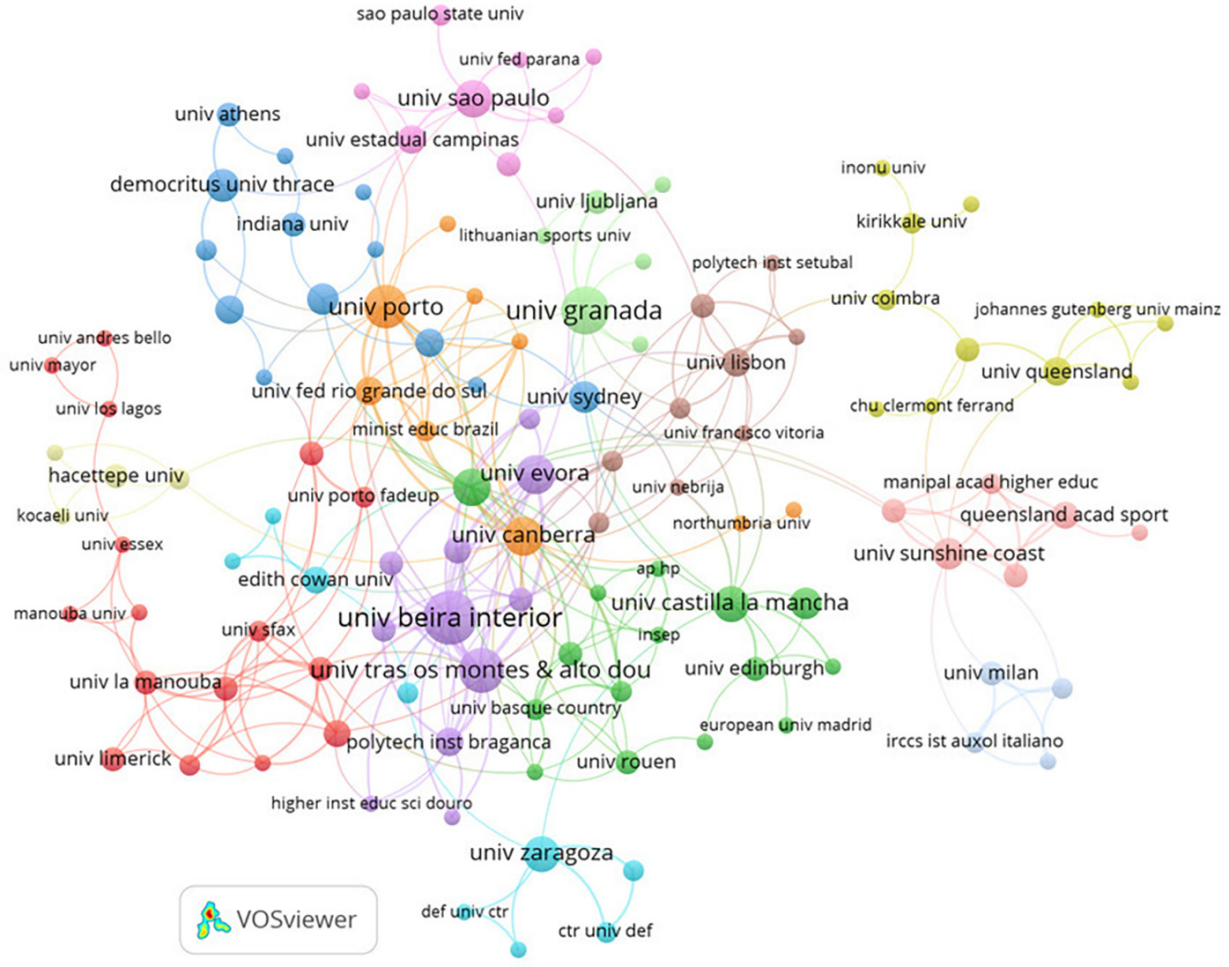
Node map for the relationship between the organization.

Table 2 considering the institutions associated with the studies, a total of 726 institutions were identified, with 29 of them with a minimum of five published studies. In this regard, the “Universidade da Beira Interior” is associated with the highest number of documents (*n* = 20). On the other hand, the “University of Copenhagen” is the organization with the highest number of citations (*n* = 619), with only three documents published. Figure 3 shows the interactions between the most prolific institutions.

**Table 2:** Most prolific affiliations considering number of publications and citations.

Affiliations	Documents	% of 460	Citations
Universidade da Beira Interior	20	4.34	410
Universidade do Porto	16	3.47	200
University of Granada	16	3.47	184
National Kapodistrian University of Athens	14	3.04	27
University of Tras os Montes Alto Douro	14	3.04	333
Instituto Politecnico de Braganca	11	2.39	164
University of Canberra	11	2.39	259
University of Evora	11	2.39	184
University of North Carolina	11	2.39	190
Australian Institute of Sport	10	2.17	282

### 3.4 Interactions between countries

**Figure 4:**
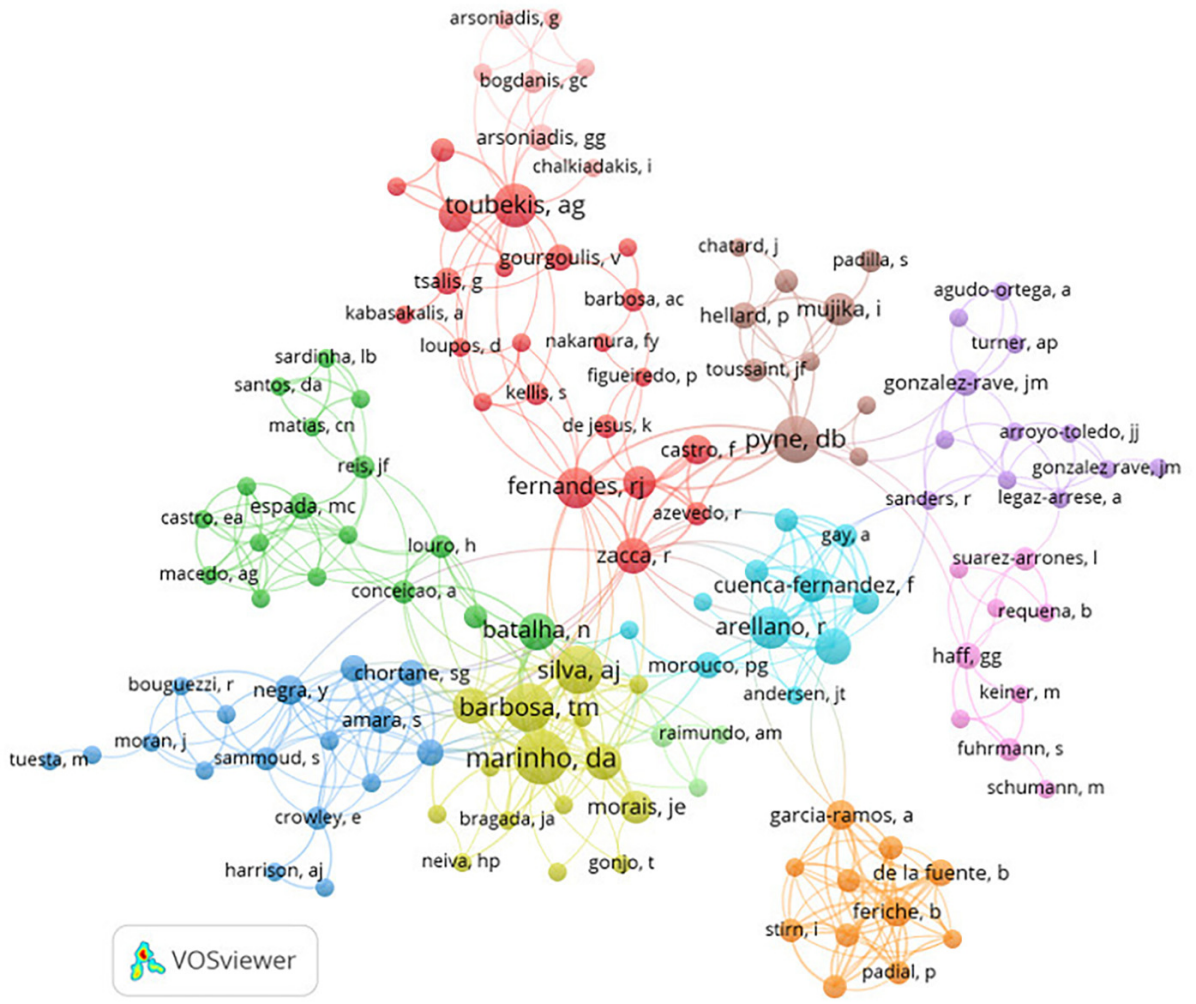
Node map regarding the relationship between the countries.

**Figure 5:**
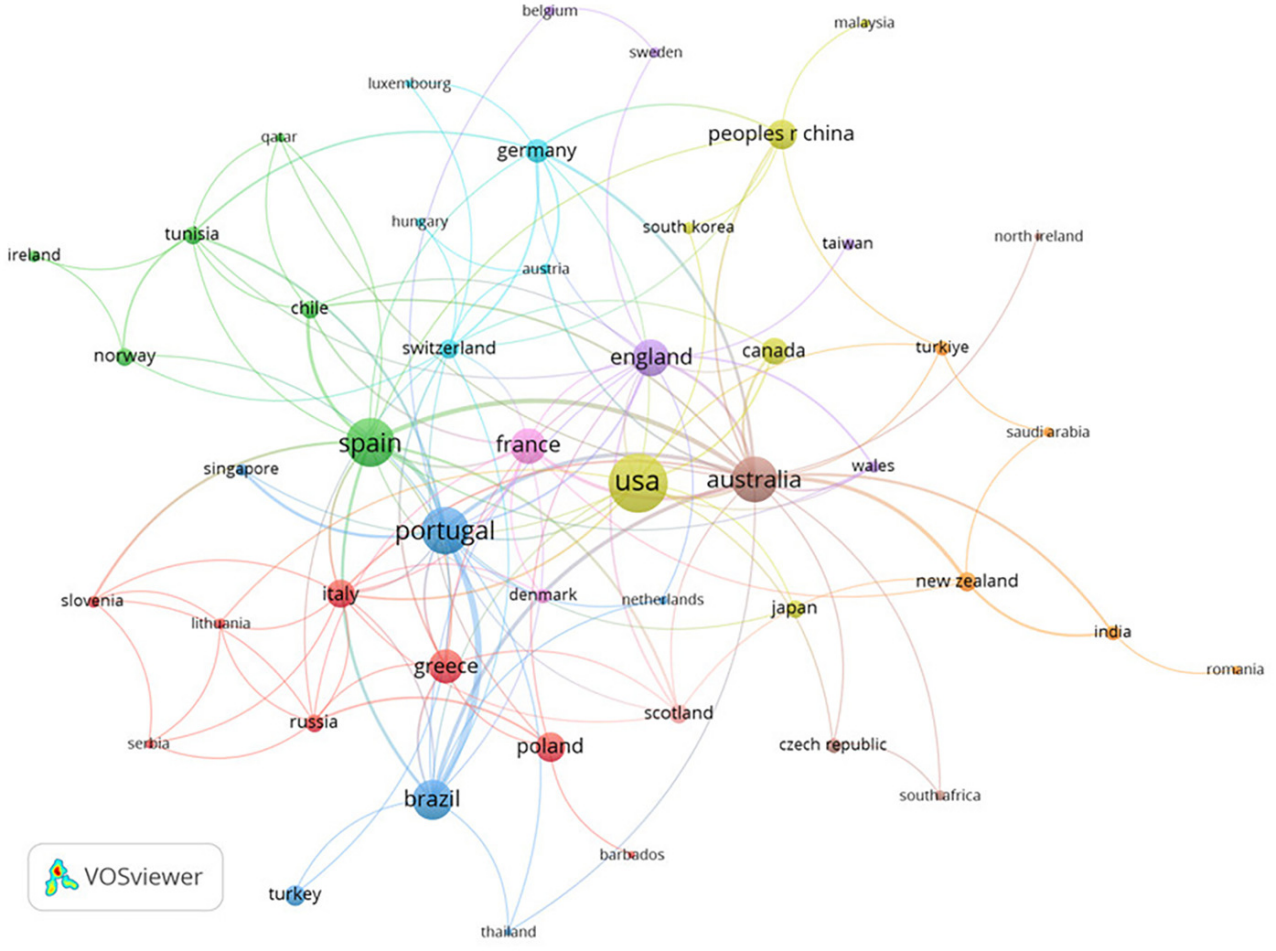
Node map of the relationship between the countries, considering the temporality.

Figures 4, 5 show that a total of 60 countries were identified, of which 10 have a minimum of 20 published documents. The United States of America is the country with the highest number of documents (*n* = 83) (Table 3).

**Table 3:** Countries with more published documents.

Country	Documents	% of 460
United States of America	83	18.04
Spain	57	12.39
Portugal	53	11.52
Australia	50	10.87
Brazil	37	8.04
England	33	7.17
France	29	6.30
Greece	27	5.87
China	20	4.34
Poland	20	4.34

### 3.5 Most prolific keywords

**Figure 6:**
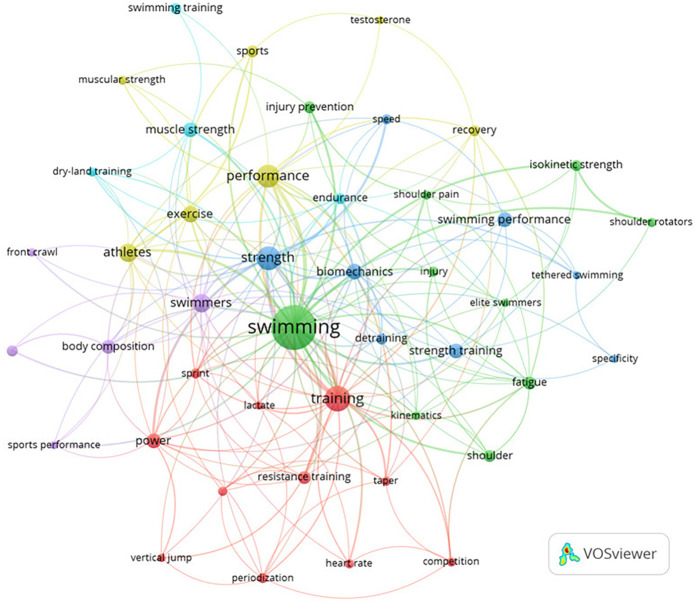
Node map for the relationship between the co-occurrences for keywords.

A total of 1,019 keywords were identified; this number relates to an average of 2.21 keywords for each document. 110 terms were identified with an occurrence of 3 words, particularly “swimming”, “training”, and “strength” the most prolific keywords with an occurrence of 128, 42, and 36, respectively (Figure 6).

**Figure 7:**
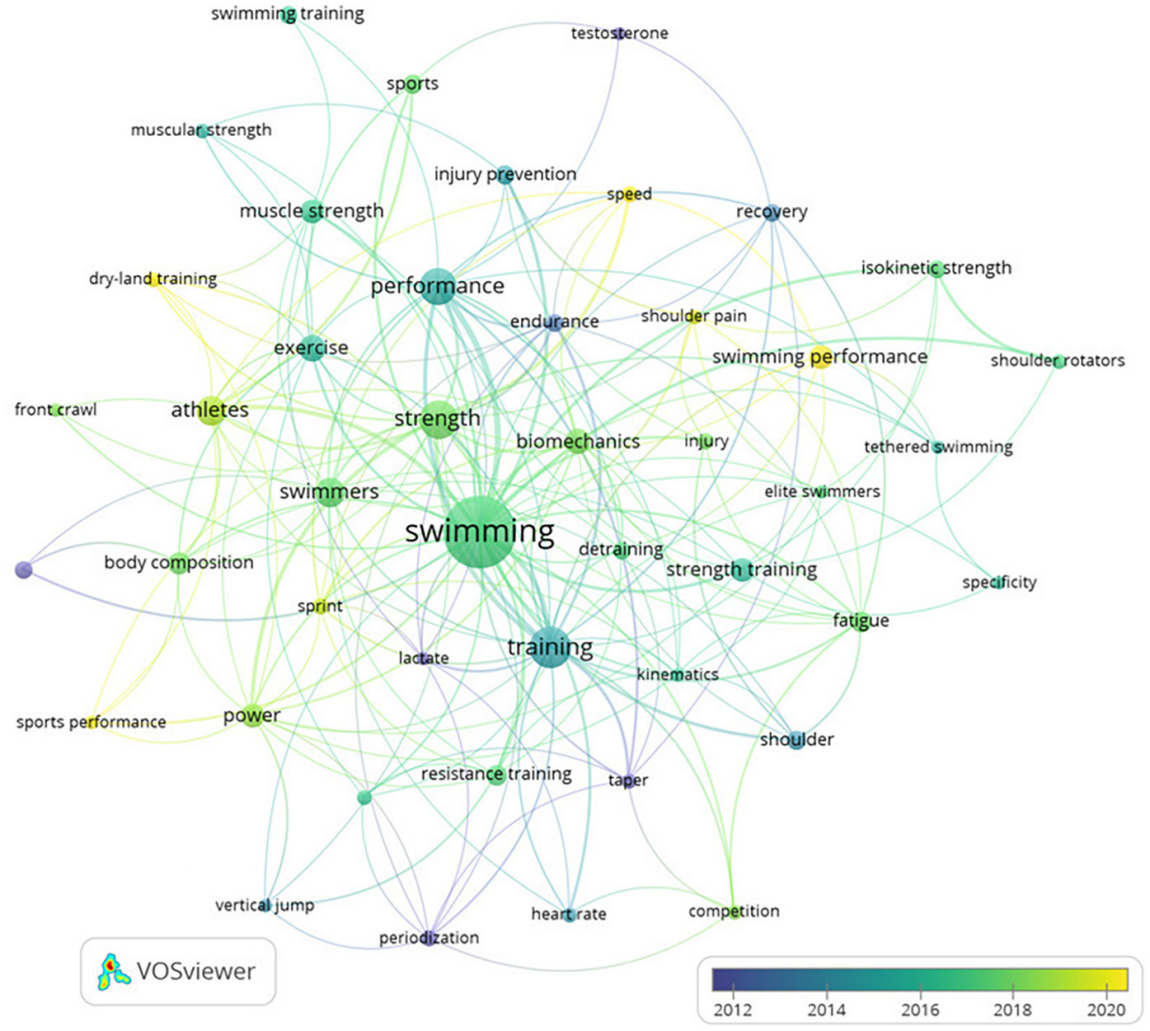
Node map for the relationship between the co-occurrences for keywords considering the timing.

Figure 7 shows the interactions between the most prolific keywords considering the timing of publication. In this case, the most recurrent terms used by the authors are “sports performance”, “speed”, and “swimming performance”. These terms exhibit a change in the scientific paradigm since the lines of research have evolved to the evaluation of swimmers' performance, considering speed as a determinant variable for a better performance.

## 4 Discussion

The purpose of this study was to conduct a bibliometric and scoping review to map the evolution of scientific literature on strength training in competitive swimmers. The primary conclusions of this scoping and bibliometric review show that, particularly after 2010, there was a notable increase in the amount of research on strength training for competitive swimmers, with a peak in publications in 2021. The University of Beira Interior and the University of Copenhagen were found to have made significant contributions to the field in terms of publications and citations, respectively. A change in the research paradigm toward variables directly related to competitive performance was reflected in the keyword analysis, which showed an evolution in the thematic focus with increasing emphasis on terms like “sports performance,” “speed,” and “swimming efficiency.”

The results showed that the first document was published in 1980. However, there was no regular publication until 1992, which was the first year with at least one document published. We identified Marinho, D.A., associated with 17 publications and “Universidade da Beira Interior” with more citations (*n* = 410), followed by the “University of Trás os Montes Alto Douro” with 333. Nonetheless, the University of Copenhagen possesses the highest citation count (*n* = 619), despite having published only three documents. The United States of America is the country with more published documents (*n* = 83), followed by Spain and Portugal, with 57 and 53, respectively. The keywords “swimming”, “training”, and “strength” were found to be the most prolific keywords.

The ramifications of these classifications in the context of ST in swimming research were examined, extending the classification within the WoS database. The classification sheds light on the study and emphasizes how multidisciplinary the field is, including connections to sports science, physiology, biomechanics, and training techniques (48).

When the participating institutions were analysed, 726 were found, indicating a wide research network. The “University of Copenhagen” has the highest number of citations (*n* = 619) with just three publications, whereas the “University of Beira Interior” stands out for having the highest number of published documents (*n* = 20). The disparity between the quantity of publications and citations seems to indicate that the productions from the University of Copenhagen are of particularly high caliber and influence, and thus, were mainly for long years the focus of citations, with other institutions such as the “University of Beira Interior” or the “University of Trás os Montes Alto Douro” associated to more recent research, years with globally more scientific production, as consequently, a more distributed citation between research. The contacts between the most active institutions are depicted in Figure 3, which shows global cooperation and information sharing important contributors, including researchers from Australia, Spain, Portugal, and Switzerland.

The present results identified 60 countries, which underlines the research topic's worldwide significance, particularly in the United States of America (*n* = 83). This country plays a pivotal role in Sports Science research, as evidenced by its high level of international cooperation (Figure 4). The increasing body of research demonstrates the value of ST for swimmers and the ongoing pursuit of better training methods (Table 3). The main themes of the study are revealed by examining the most popular terms (Figure 6). Based in the findings of this study, in our perspective, the distinguished factor regarding scientific production in strength training in competitive swimmers is the network between authors and institutions, not the international level of athletes’ or results in international events such as the Olympic Games, in which, countries such as the United States of America, China and Australia have dominated over the years.

A variety of methods and interests within the area are suggested by the discovery of 1,019 keywords, with an average of 2.21 per document, that lead us to observe a range of approaches and interests within the field (48). The chronological history of the keywords (Figure 7) indicates a shift in the scientific perspective. Terms such as “sports performance,” “speed,” and “swimming performance” have gained popularity due to the increased emphasis on assessing swimmers’ performance and the importance of speed as a determining factor. This change in focus can be connected to the creation of more precise measurement instruments (8), and the growing demand for high-performance results in competitive sports, something Morouco et al. (49) previously stated whether ST improves swimming performance and how such training should be planned to maximize performance are not well understood from a scientific standpoint.

The results of the research have significant implications for the training of swimmers. The importance of ST has been recognized, and the advances in performance evaluation methods have allowed for the creation of more customized and effective training programs. Collaboration between academic institutions and foreign organizations regarding ST in swimming facilitates the sharing of best practices and expertise (50).

These findings demonstrate a maturing understanding of strength training in the context of competitive swimming, in addition to reflecting the rise in scientific output on the topic. The fact that the most productive nations include the USA, Portugal, and Spain indicates that efforts to comprehend the mechanisms underlying elite performance are becoming more widespread. However, the high concentration of inter-institution collaborations suggests that networks of cooperation are crucial to scientific advancement in this field. The increasing emphasis on words like “speed” and “performance” as key terms points to an effort by the scientific community to convert strength training's benefits into quantifiable and objective gains. This marks a significant shift from studying ST as a stand-alone element to incorporating it into applied and deterministic performance models. Furthermore, the findings highlight the necessity of standardizing procedures to more effectively compare effects across studies and conducting longitudinal research to examine the long-term effects of ST. These results highlight how crucial it is to incorporate strength training into swimmers' regimens while honoring the unique characteristics of each swimmer and the particulars of the events. As a result, bibliometric analysis offers a strategic roadmap to direct future research, enabling us to pinpoint areas of application, possible partnerships, and gaps that have the biggest effects on sports practice.

In summary, this review is important since it thoroughly examines and compiles the literature showing the substantial impact of ST on swimmers. Additionally, it shows how the scientific community is becoming more interested in this research topic. These results offer direction for further research and targeted interventions meant to persuade all coaches not to fear swimmers' dry-land training, targeting interventions designed to persuade all coaches that swimmers' dry-land training is not a source of apprehension and may serve as a crucial factor in enhancing swimming performance. One of the limitations of the study is the selection of keywords, and another, the consideration of only manuscripts written in English. Aiming the correct progress of the study, we selected those words that are closest to the topic, eliminating biases in the results, therefore, ensuring that the studies identified were specifically related to the study topic. Future research may also use different databases, not only WoS and consider different areas closely related to swimming performance.

## 5 Conclusion

The findings revealed that the initial document was published in 1980. However, there were no regular publications of such documents until 1992, which marked the first year with at least one document published.

The country with the highest number of documents published was the United States of America, with a total of 83. It should be emphasized that Portuguese authors and institutions are among those who have focused the most on this research topic.

These results highlight that bibliometric analysis provides pertinent information, very useful regarding research trends and networks, aiming for future research on the topic of strength in swimmers.
